# 114. Optimization of Inpatient Antibiotic Use via an Electronic Antimicrobial Stewardship Module and an Infectious Diseases Pharmacy Resident

**DOI:** 10.1093/ofid/ofab466.316

**Published:** 2021-12-04

**Authors:** Victor Chen, Lauren Allen, Hongkai Bao, Kelsie Cowman, Priya Nori, Priya Nori, Yi Guo

**Affiliations:** 1 UC San Diego Health, San Diego, California; 2 Montefiore Medical Center, Newark, New York; 3 Montefiore Medical Center and Albert Einstein College of Medicine, New York, NY; 4 Montefiore Medical Center/Albert Einstein College of Medicine, Bronx, NY; 5 Montefiore Medical Center, Albert Einstein College of Medicine, Bronx, NY

## Abstract

**Background:**

Antibiotic resistance is a public health crisis and antimicrobial stewardship (AMS) pharmacists serve a crucial role in preventing inappropriate use. At Montefiore Medical Center (1,500-bed hospital), a new electronic medical record AMS module was implemented with assistance from an infectious diseases (ID) pharmacy resident in October 2020. The module utilizes a dynamic scoring system to assist in prioritizing interventions, including bug-drug mismatches, insufficient coverage, or de-escalation. The AMS module is operationalized by ID pharmacists during the week and an ID pharmacy resident every other weekend. The objective of this study was to assess the impact of an ID pharmacy resident performing AMS module interventions on broad spectrum antibiotic use.

**Methods:**

An observational study of AMS module interventions on antibiotic use (AU) in days of therapy per 1,000 days present and standardized antimicrobial administration ratio (SAAR) was performed. AU data for piperacillin-tazobactam (P/T) and SAAR prior to (October 2019– December 2019) and after (October 2020 – December 2020) the integration of an ID pharmacy resident and the AMS module was compared. Additional data collected included total number and type of interventions.

**Results:**

A total of 539 interventions were made by AMS pharmacists and 36.5% of these were completed by the ID pharmacy resident. Across 6 different units, there was a statistically significant decrease in the SAAR for broad spectrum antibacterial agents (Figure 1), and a decrease of at least 10% in P/T use during the two different time periods (Table 1). An estimated P/T cost reduction of 26% of (&48,708 to &36,235.80) was observed. AMS pharmacists made 63 interventions in respective units. The top three intervention types were dose/frequency/duration recommendations, pharmacokinetic vancomycin dosing/monitoring, and de-escalation. The acceptance rate of interventions was 99% (534 accepted interventions/539 total interventions).

Figure 1. SAAR Comparison of Broad-Spectrum Agents

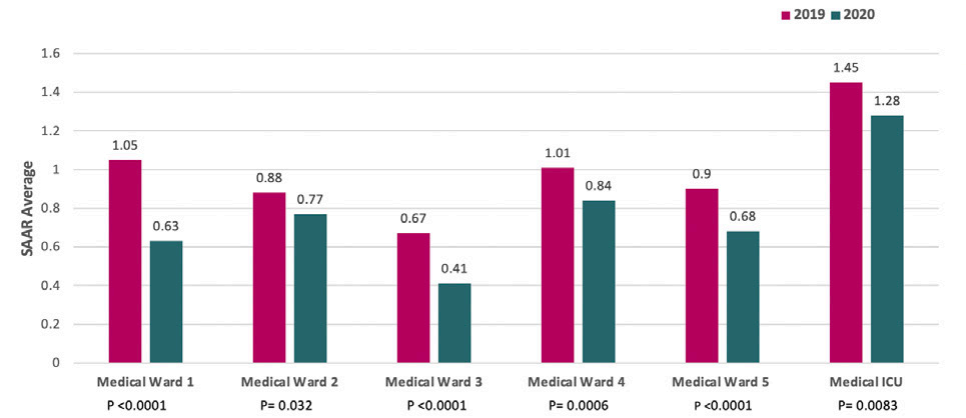

Table 1. AU Rate of Piperacillin-tazobactam

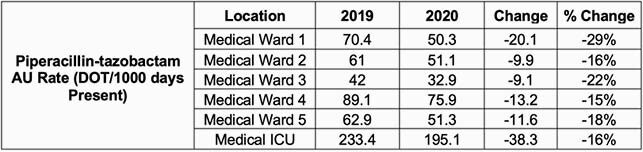

**Conclusion:**

Overall, there was a statistically significant impact on SAARs and a >10% change in P/T AU rate with an estimated cost reduction >25% on select units after implementation of the AMS module with an ID pharmacy resident.

**Disclosures:**

**Kelsie Cowman, MPH**, **Merck** (Research Grant or Support) **Priya Nori, MD**, **Merck** (Grant/Research Support) **Priya Nori, MD**, Nothing to disclose **Yi Guo, PharmD, BCIDP**, **Merck** (Research Grant or Support)

